# Lightning Burns and Electrical Trauma in a Couple Simultaneously Struck by Lightning

**DOI:** 10.5811/cpcem.2017.4.33706

**Published:** 2017-07-17

**Authors:** Stephanie A. Eyerly-Webb, Rachele Solomon, Seong K. Lee, Rafael Sanchez, Eddy H. Carrillo, Dafney L. Davare, Chauniqua Kiffin, Andrew Rosenthal

**Affiliations:** Memorial Regional Hospital, Division of Acute Care Surgery and Trauma, Hollywood, Florida

## Abstract

More people are struck and killed by lightning each year in Florida than any other state in the United States. This report discusses a couple that was simultaneously struck by lightning while walking arm-in-arm. Both patients presented with characteristic lightning burns and were admitted for hemodynamic monitoring, serum labs, and observation and were subsequently discharged home. Despite the superficial appearance of lightning burns, serious internal electrical injuries are common. Therefore, lightning strike victims should be admitted and evaluated for cardiac arrhythmias, renal injury, and neurological sequelae.

## INTRODUCTION

In the United States, the National Weather Service estimates the odds of being struck by lightning in a given year as approximately one in a million. In Florida, the heat and humidity of the subtropical climate creates optimal conditions for thunderstorms; the density of lightning strikes is approximately 30 strikes/km^2^ per year, relatively high on the global scale and the highest in the U.S.^1^ The risk presented by this high strike density in conjunction with the large population density and favorable climate for outdoor and water activities has earned Florida the reputation as the “Lightning Capital of the United States.”^2^,^3^ Although being struck by lightning is considered an uncommon occurrence, Florida has more than twice as many lightning-related casualties and fatalities than any other state and averages approximately 10 fatalities per year.^3^,^4^

While several published case reports describe the injuries of individuals struck by lightning, this report presents a couple that was simultaneously struck by lightning while walking arm-in-arm in the rain holding an umbrella between them. Their unique lightning burns and treatment are discussed.

## CASE PRESENTATION

### Patient 1

A 40-year-old male was struck by lightning while walking in a rainstorm holding an umbrella in an open parking lot. He presented with superficial and partial thickness cutaneous injuries. He recalled falling to his knees and losing sensation in his left leg and subsequently suffered loss of consciousness for a very short period of time post-strike.

The patient presented, alert, oriented, and responsive with a Glasgow Coma Score (GCS) of 15, but complained of diffuse pain. His vitals were normal and he was in normal sinus rhythm (NSR) with no heart murmurs. A focused assessment with sonography for trauma (FAST) and abdominal examination were negative and there was no evidence of compartment syndrome or internal injury. Chest and pelvic radiography as well as computed tomography (CT) of the head and cervical spine were normal.

**Image 1:**
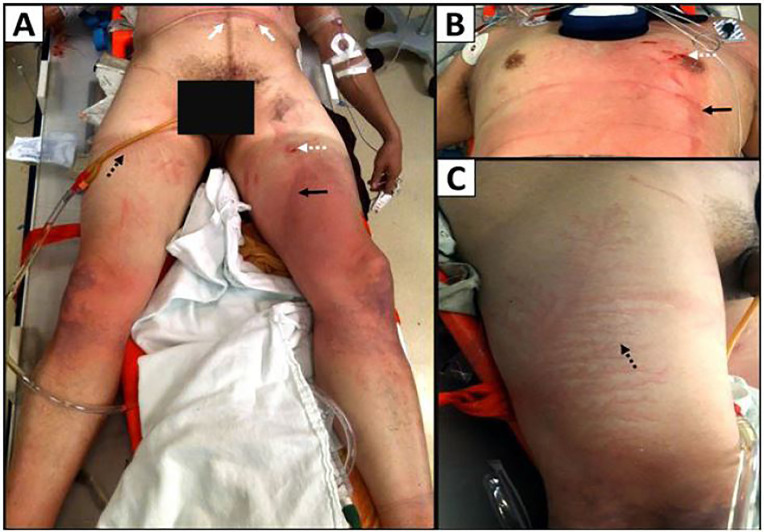
Cutaneous injuries on patient one: (A) Small superficial thermal burns where the patient’s belt buckle contacted the skin (white arrows). Lichtenberg figure lesions on anterior right thigh (black dotted arrow). Superficial punctate burn on left anterior thigh (white dotted arrow). Dark linear flash burn on left anterior thigh (black arrow). Dark purple ecchymosis is visible on the patient’s knees from his fall to the ground after the strike; (B) Superficial linear flash burn (black arrow), punctate burns (white dotted arrow), and small abrasions over left nipple; (C) Lichtenberg figure lesions on the lateral right thigh (black dotted arrow).

He sustained multiple burn injuries, including superficial burns on the anterior trunk and legs (Image 1). Small abrasions were also observed above the scrotum area and the left nipple, and partial thickness punctate burns were observed on the anterior left thigh and over the left nipple (Images 1A and 1B). Minor superficial thermal contact burns were observed below his belt buckle (Image 1A). Superficial flash burns were also observed on the patient’s trunk and anterior thighs, and ecchymosis was observed on the patient’s knees, resulting from his fall to the ground. Distinct Lichtenberg figure lesions were observed on the anterior right thigh (Image 1A) as well as on the right and left lateral thighs (Image 1C). The patient also presented with an open wound on the left lateral ankle; his shoe had been burnt and blown off by the strike.

The patient tested positive for blood (3+) and albumin (1+) in the urine, and was diagnosed with myoglobinuria and admitted to the intensive care unit for hemodynamic monitoring and resuscitation. The patient’s troponin-T, creatinine kinase myocardial b fraction (CK-MB), and total CK levels were monitored to identify any cardiac or global muscle damage. His total CK levels were elevated (1,654 unit/L) upon arrival and trended down over his 68-hour hospital course. Troponin-T and CK-MB levels remained normal. The patient remained neurologically intact with no sequelae and was subsequently discharged home.

### Patient 2

**Image 2:**
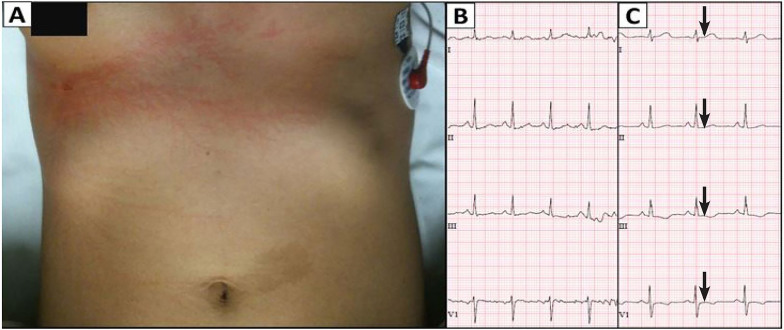
(A) Lichtenberg figure lesions on the chest; (B) Normal electrocardiogram upon admittance; (C) Subsequent electrocardiogram nine hours after admittance demonstrating abnormal ST-segments and inverted T-waves (arrows).

A 30-year-old female was simultaneously struck by lightning while holding the umbrella with Patient 1. She sustained small partial thickness burns to the right cheek and the inside of her right index finger where she was holding the umbrella. Lichtenberg figure lesions were observed on the patient’s chest (Image 2A). The patient was alert and oriented with a GCS of 15 on arrival.

She presented with normal vital signs. There was no evidence of compartment syndrome and a FAST scan and abdominal examination were normal. Radiography of the chest and pelvic area as well as CTs of the head and cervical spine were normal.

The patient was mildly tachycardic (~100 beats per minute) on arrival with complaint of “pressure”-like discomfort in her chest despite having a normal rhythm (Image 2B), no heart murmurs, and normal troponin-T and CK-MB levels. She was admitted to the hospital for monitoring. Serum labs at six and nine hours showed slightly elevated troponin-T (troponin-T_6hr_ = 0.05 ng/mL, troponin-T_9hr_ = 0.02 ng/mL) and CK-MB (CK-MB_9hr_ = 5.6 ng/mL) levels and a follow-up electrocardiogram (ECG) exhibited an abnormal ST-segment, all of which could be predictive of an adverse cardiac event (Image 2C). Her total CK-MB levels returned to normal range within 24 hours, and the ECG returned to normal before discharge. Her CK total levels were slightly elevated (>300 unit/L) but returned to normal. She remained neurologically intact and was discharged with no sequelae after 66 hours.

## DISCUSSION

A lightning strike creates a short duration (10–100 ms) discharge circuit (30,000–50,000A) between the clouds and strike location.^5^–^7^ Peak discharge point is typically 2–5 cm in diameter and spreads radially across the strike surface.^8^ Direct lightning strikes are uncommon and often fatal and comprise approximately 5% of strike events.^6^,^9^ The vast majority of lightning-related injuries are indirect.^6^,^9^ Indirect injury methods include the following: 1) side flash or “splash” contact injury, where the victim is in contact with the struck object, 2) ground current, where the current passes across the victim from a nearby ground strike point, and 3) blast injury from sonic-wave thunder, which includes primary injury methods such as sonic rupture of tympanic membranes or tertiary injury methods such as blunt trauma if the victim falls or is thrown.^5^,^7^,^9^ Ultimately 10–30% of lightning strike victims die, and most deaths are either immediate or due to cardiac or respiratory arrest.^5^,^7^,^9^,^10^

CPC-EM Capsule

What do we already know about this clinical entity?

Lightning injuries, although rare, are serious and life-threatening. While risk is somewhat geographical, lightning-related injuries have the potential to affect large populations.

What makes this presentation of disease reportable?

This report illustrates the case of a couple simultaneously struck by lightning, which has rarely been reported.

What is the major learning point?

A single lightning strike can inflict both external and internal injuries on multiple patients; all injuries require timely diagnosis and treatment.

How might this improve emergency medicine practice?

Although most clinicians will never treat a patient struck by lightning, they must be prepared to recognize and treat this potentially lethal injury.

Lightning strike victims often present with cutaneous injuries, which include linear or flash burns, punctate burns, thermal burns, or Lichtenburg figure lesions. Linear flash burns are in effect scald burns caused by the rapid heating and evaporation of water from the surface of the skin. These burns are often superficial and occur where there is moisture (sweat, rain) on the skin.^5^,^10^ Punctate burns are small, circular burns that can be superficial or full thickness. They are typically very small and do not require medical treatment.^7^,^11^ Patients struck by lightning may also suffer serious thermal burns from metallic accessories or objects contacting their skin. The short contact duration (tens of milliseconds) and relatively high resistance of the epidermis often prevents significant direct tissue heating damage due to the electrical current alone; but the strike can quickly heat any metallic objects, such as a necklace or belt buckle, that in turn can cause severe thermal contact burns.^7^,^12^–^14^ Thermal burns may also occur if a victim’s clothing ignites.

A less common cutaneous injury unique to lightning strike victims are unusual superficial lesion patterns known as Lichtenberg figures. They are also known as arborization or “fern,” “feathering” lesions based on their unique fractal appearance.^4^–^7^ Lichtenburg figure lesions are exclusively caused by lightning and are not technically burns, as they are not thermal injuries and the dermis and epidermis remain intact.^4^,^10^ Rather, the lightning energy spreads in a fractal pattern across the skin, which causes a transient extravasation of blood and subsequent discoloration in the subcutaneous tissue.^4^,^10^ These dramatic marks typically fade within days as the tissue returns to normal. Overall, the short duration of the lightning strike and high resistance of the skin results in minimal cutaneous damage; lighting burns and lesions are typically minor, superficial, and require little treatment.^5^,^6^,^10^,^14^

Despite the superficial appearance of burns on lightning strike victims, occult internal electrical injuries can be deadly and do not correlate with the severity of cutaneous injuries. Internal muscles and organs are highly conductive and can be severely damaged by the energy of the strike.^10^,^14^ For example, immediate cardiorespiratory arrest or ventricular fibrillation cardiac arrest are the primary causes of death for lightning strike victims.^10^,^15^ Lightning strikes can also cause acute cardiovascular conditions such as arrhythmias, myocardial ischemia, and even myocardial contusion.^7^,^14^–^17^ Monitoring the patient’s ECG and cardiac enzymes such as troponin-T and CK-MB may be useful for determining the extent of myocardial damage inflicted by the electrical trauma.^14^–^16^,^18^

In addition to cardiac complications, 3–15% of lightning strike victims may develop acute renal failure.^19^ The electrical trauma of the strike can cause severe muscle damage along the conduction pathway; when this necrotic muscle tissue breaks down it releases excessive amounts of toxic muscle-cell components, such as CK, into circulation.^19^ This can lead to myoglobinuria and potentially catastrophic renal failure.^7^,^15^,^19^ Patient total CK levels have been shown to predict the amount of muscle injury and should be monitored to prevent lasting renal issues.^18^,^19^ Sensory damage (eye, ear) and neurological sequelae, such as hypoxic ischemic neuropathy, intracranial hemorrhage, motor neuron disease, and movement disorders, are also common in strike victims.^5^–^10^,^14^,^20^ Thorough follow-up for all these potential complications is recommended as approximately 75% of strike survivors are at risk of long-term or permanent sequelae.^5^,^10^

## CONCLUSION

The National Weather Service advises everyone to immediately seek shelter if lightning and thunder are present, and asserts that remaining outside during a storm places individuals at greater risk. Prevention measures should be taken seriously in Florida, which consistently has the largest number of lightning strike casualties and fatalities in the nation due to frequent tropical storms and enthusiasm for outdoor leisure activities. If struck by lightning, an individual can suffer a multitude of serious external and internal injuries. Strike victims should be admitted and evaluated for long-term issues such as cardiac arrhythmias, renal injury and myoglobinuria, sensory damage, and neurological sequelae.

## References

[1] Christian HJ, Blakeslee RJ, Boccippio DJ (2003). Global frequency and distribution of lightning as observed from space by the Optical Transient Detector. J Geophys Res.

[2] Jensenius JS (2015). A detailed analysis of lightning deaths in the United States from 2006 through 2014.

[3] Curran EB, Holle RL, López RE (2000). Lightning casualties and damages in the United States from 1959 to 1994. J Climate.

[4] Resnik BI, Wetli CV (1996). Lichtenberg Figures. Am J Forensic Med Pathol.

[5] Ritenour AE, Morton MJ, McManus JG, Barillo DJ, Cancio LC (2008). Lightning injury: a review. Burns.

[6] Cooray V, Cooray C, Andrews CJ (2007). Lightning caused injuries in humans. J Electrostat.

[7] O’Keefe Gatewood M, Zane RD (2004). Lightning injuries. Emerg Med Clin North Am.

[8] Bier M, Chen W, Bodnar E, Lee RC (2005). Biophysical injury mechanisms associated with lightning injury. Neurorehabilitation.

[9] Pfortmueller CA, Yikun Y, Haberkern M (2012). Injuries, sequelae, and treatment of lightning-induced injuries: 10 years of experience at a Swiss trauma center. Emerg Med Int.

[10] Forster SA, Silva IM, Ramos MLC (2013). Lightning burn - Review and case report. Burns.

[11] Fahmy FS, Brinsden MD, Smith J (1999). Lightning: the multisystem group injuries. J Trauma Acute Care Surg.

[12] Herrero F, Garcia-Morato V, Salinas V (1995). An unusual case of lightning injury: a melted silver necklace causing a full thickness linear burn. Burns.

[13] Bayramoglu AA, Uzkeser M, Kamaci U (2012). Lichtenberg Figures. Duzce Medical Journal.

[14] Cooper MA (1995). Emergent care of lightning and electrical injuries. Semin Neurol.

[15] McIntyre WF, Simpson CS, Redfearn DP (2010). The lightning heart: a case report and brief review of the cardiovascular complications of lightning injury. Indian Pacing Electrophysiol J.

[16] Lichtenberg R, Dries D, Ward K, Marshall W, Scanlon P (1993). Cardiovascular effects of lightning strikes. J Am Coll Cardiol.

[17] Dundon BK, Puri R, Leong DP, Worthley MI (2009). Takotsubo cardiomyopathy following lightning strike. BMJ Case Reports.

[18] Teodoreanu R, Popescu S, Lascar I (2014). Electrical injuries. Biological values measurements as a prediction factor of local evolution in electrocutions lesions. J Med Life.

[19] Okafor UV (2005). Lightning injuries and acute renal failure: a review. Ren Fail.

[20] Cherington M, Yarnell P, London S (1995). Neurologic complications of lightning injuries. West J Med.

